# State-of-the-Art Biosensors in China

**DOI:** 10.3390/bios16060328

**Published:** 2026-06-09

**Authors:** Nan Xiang, Chun-Yang Zhang

**Affiliations:** 1 School of Mechanical Engineering, Jiangsu Key Laboratory for Design and Manufacturing of Precision Medicine Equipment, Southeast University, Nanjing 211189, China; nan.xiang@seu.edu.cn; 2 School of Chemistry and Chemical Engineering, State Key Laboratory of Digital Medical Engineering, Southeast University, Nanjing 211189, China

Biosensors are analytical devices that integrate a biological or synthetic recognition element (e.g., nucleic acids, enzymes, and antibodies) with a transducer. Upon interaction with the target analyte, the biorecognition receptor generates a response that can be converted by the transducer into a measurable signal, such as optical, electrochemical, or piezoelectric readouts [1]. The rapid advancement of biosensors has recently attracted increasing interest among researchers worldwide, driven by the vast potential of these devices across diverse fields, including health, medicine, agriculture, industry, defense, food safety, and environmental monitoring [2]. Compared with conventional analytical techniques, biosensors offer significant advantages, such as high specificity and sensitivity, rapid detection, low cost, portability, user-friendliness, and ease of specimen collection.

To date, biosensors have been widely employed for a range of applications, such as the monitoring and diagnosis of diseases (e.g., cancer, infectious diseases, and heart-related diseases) through biomarkers in biofluids and the identification of pollutants (e.g., pesticides, antibiotic residues, and heavy metals) in environmental and food samples [3,4]. Various innovative sensing principles, including colorimetric, electrochemical, fluorescence, luminescence, surface-enhanced resonance Raman scattering (SERS), and nanomaterial-based or aptamer/antibody-based sensing, have been reported [5,6]. In parallel, rapid and sensitive biosensing techniques have been developed, such as microfluidic chips, single-molecule imaging, smartphone sensing, wearable devices, and point-of-care testing devices [7,8]. Moreover, when integrated with emerging communication technologies and deep/machine learning approaches, biosensors become even more facile, useful, and accessible, greatly enhancing their applicability to customized healthcare and environmental monitoring systems [9,10].

We believe that biosensors will continue to propel transformative progress in both diagnostic and monitoring practices. Our Special Issue, entitled “State-of-the-Art Biosensors in China (Volume II)”, is devoted to the most recent progress and innovations in the development of biosensors in China. We invited original and review articles from researchers across different disciplines that address these cutting-edge themes. A total of 13 outstanding papers (including seven research articles and six reviews) were published in our Special Issue. Next, we will briefly introduce these papers.

Liu et al. (contribution 1) reported a ratiometric electrochemical biosensor for the detection of interleukin-6 (IL-6). The biosensor employs an electropolymerized methylene blue (MB) layer on screen-printed carbon electrodes as an internal reference signal probe and utilizes carboxylated multi-walled carbon nanotubes to enhance the electrochemically active area of electrodes. IL-6-specific aptamers are immobilized on the electrode surface via the Schiff base reaction. Upon the binding of IL-6, the current from the [Fe(CN)6]3-/4- redox probe decreases, while the MB signal remains stable, enabling self-calibrated quantification. The sensor demonstrated a wide linear range of 0.001~1000.0 ng/mL and a low detection limit of 0.54 pg/mL and was successfully applied to serum sample analysis.

Gao et al. (contribution 2) developed a multi-channel electrochemical sensing system integrated with electrochemical analysis techniques, microelectronic design, and nanomaterials for the simultaneous analysis of key urine biomarkers. The system features dedicated electrodes for creatinine, uric acid, and pH, with each channel exhibiting a defined linear detection range. The detection ranges are 10 nM to 100 μM for creatinine, 100 μM to 500 μM for uric acid, and 4 to 9 for pH. The hardware incorporates stored calibration equations, and a smartphone application (WeChat mini-program) enables wireless data transmission and real-time display. This design aims to facilitate point-of-care and at-home intelligent medical monitoring.

Shen et al. (contribution 3) provided a comprehensive review on the recent development of electrochemical immunosensors for organophosphate pesticide detection. The review discusses the fundamental principles of electrochemical immunosensors and focuses on the strategies of novel nanomaterials for signal amplification, enzyme-based methods, and innovative electrode architectures to enhance sensitivity and selectivity. The integration of electrochemical immunosensors into microfluidic devices and the application of screen-printed and disposable electrodes for field-deployable testing are discussed. Emerging trends, including the use of artificial intelligence and aptamer-based sensors and the integration of multi-analyte detection platforms and the Internet of Things, as well as challenges for commercialization, are highlighted.

Li et al. (contribution 4) investigated a binary surfactant mixture of sodium dodecyl benzene sulfonate (SDBS) and fatty alcohol-polyoxyethylene ether (AEO) as a potential environmentally friendly replacement for Triton X-100 in biosensor manufacturing. The study comprehensively compared the surface activity, micelle formation, dynamic adsorption, foaming, emulsifying, and cell permeability properties of the SDBS-AEO mixture with those of TX-100. Theoretical and experimental results indicate that a properly formulated SDBS-AEO mixture exhibited comparable performance to TX-100, offering a promising alternative to address ecological concerns associated with TX-100 degradation products.

Jia et al. (contribution 5) designed a microfluidic sensor integrated with surface-enhanced Raman spectroscopy (SERS) and a cactus-like array substrate (CAS) for the sensitive detection of prostate cancer-derived exosomes. The core of the sensor is a three-dimensional CAS constructed from self-assembled polystyrene microspheres, which provide high density of SERS “hot spots”. Using a sandwich assay format with aptamers targeting exosome surface markers (EpCAM and CD63), the researchers showed that the sensor achieved a wide linear detection range of of 105 particles/μL to 1 particle/μL and an ultra-low limit of detection (1 particle/μL) for exosomes in Lymph Node Carcinoma of the Prostate, demonstrating potential for the early diagnosis of prostate cancer using their new CAS integrated sensors.

Wu et al. (contribution 6) designed and synthesized a mitochondria-targeted, ratiometric fluorescent probe (MOBDP-I) for the detection of peroxynitrite (ONOO-) in the mitochondria of inflammatory cells and model mice. The probe was constructed by linking a BODIPY fluorophore to an indole-salt mitochondrial targeting moiety via a carbon–carbon double bond (C=C). Oxidation and cleavage of this C=C bond by ONOO- induced an 18-fold enhancement in fluorescence at 510 nm and a significant fluorescence decrease at 596 nm. The probe was successfully applied to image ONOO- in the mitochondria of inflammatory cells and in mouse models of rheumatoid arthritis, peritonitis, and brain inflammation, serving as a tool to study oxidative stress in inflammation.

Quan et al. (contribution 7) developed a novel electrochemical glucose sensor based on a dual redox mediator system comprising 1,10-phenanthroline-5,6-dione (PD) and hexaammineruthenium(III) chloride (Ru(III)). In this system, the synergistic effect between PD and Ru(III) is harnessed to efficiently facilitate electron transfer between the enzyme’s active center and the electrode. This synergistic mechanism enables efficient electron transfer, resulting in a sensor with a wide linear range from 0.01 to 38.6 mmol/L, a high sensitivity of 38 μA·L/(mmol·cm^2^), and a low detection limit as low as 7.0 μmol/L. The sensor’s accuracy was validated through recovery tests in human blood samples. The glucose recovery rate was between 99.5% and 107%, with a relative standard deviation of less than 3.2%, showing high potential for practical applications in glucose monitoring.

Zheng et al. (contribution 8) reviewed recent research progress in the detection of small molecules using aptamer-based SERS techniques. The review introduces the fundamental principles of aptamers and SERS and summarizes their combined application in analyzing pesticides, antibiotics, and toxins. The integration leverages the high specificity of aptamers and the exceptional sensitivity of SERS, enabling trace analysis in fields such as food safety, environmental monitoring, and biomedicine. Future prospects for this synergistic technology are also discussed.

Jin et al. (contribution 9) reviewed the application of novel luminescent semiconductor materials, namely, lead halide perovskites and copper iodide hybrids, in optical oxygen sensing. The review begins with an introduction to traditional oxygen sensing mechanisms and materials. It then focuses on the oxygen sensing principles, performance, and applications of these emerging luminescent semiconductor materials, perovskite metal halides, and copper iodide hybrid materials. The adjustable optical properties and stimulus-responsive characteristics of these materials present great potential for developing advanced optical oxygen sensors.

Du et al. (contribution 10) provided a review of recent advancements in flow cytometry both worldwide and in China after 2020, focusing on instrumentation and technical progress. The review investigates state-of-the-art instruments of various types, including spectral, mass, imaging, nano-, and label-free flow cytometry. The cutting-edge technical developments aimed at achieving higher throughput, higher dimensionality, and enhanced performance are summarized. Finally, the review presents future directions for flow cytometry, with the expectation that different types will each develop along their own trajectory, gaining better performance and finding wider application in diverse areas.

Cui et al. (contribution 11) reviewed the research progress in China on electrochemical biosensors for disease detection. The article systematically categorizes and summarizes biosensors developed in China for diagnosing cancers, infectious diseases, inflammations, and neurodegenerative disorders. The working principles, classifications, key materials, and preparation techniques of electrochemical biosensors are discussed. The review also addresses the main challenges hindering the advancement of electrochemical biosensors in China and explores promising future directions for their development.

Shen et al. (contribution 12) reviewed the synthesis and biomedical applications of biodegradable nanocarriers based on polylactic acid-hyperbranched polyglycerol (PLA-HPG) copolymers. The review summarizes various synthetic strategies for PLA-HPG and the fabrication of corresponding nanoparticles, highlighting their physicochemical and biocompatible properties. Applications in skin surface delivery, wound dressing, and in vivo drug delivery are introduced. Finally, thoughts on possible directions for advancing this nanoplatform in the years ahead are presented.

Qin et al. (contribution 13) developed a highly sensitive fluorescence anisotropy (FA) assay for the detection of hepatitis B virus DNA (HBV-DNA). The method employs a dual-signal amplification strategy combining the trans-cleavage activity of the CRISPR-Cas12a system and the large molecular size of DNA tetrahedron (TDF) assemblies. The CRISPR-Cas12a system is activated by target DNA to cleave reporter probes, while the TDF assemblies provide a high and stable initial FA background. This combination results in a significant FA change, enabling detection with a wide linear range of 0.5–9 nmol/L and a low limit of detection of 48 pmol/L. The assay demonstrated excellent selectivity and was successfully applied to detect HBV-DNA in human serum.

Our Special Issue, with 13 outstanding contributions, is presented as a small step towards the development of biosensors for various biomedical applications in China.
